# Avoiding routine gastric residual volume measurement in neonatal critical care (the neoGASTRIC trial): study protocol for a multi-centre, unblinded, randomised, controlled trial

**DOI:** 10.1186/s13063-025-09403-7

**Published:** 2026-01-08

**Authors:** Elizabeth Nuthall, Amy Rodriquez, Iza Andrzejewksa, Zainab Aslam, Cheryl Battersby, Catherine Beesley, Christina Cole, Helen Campbell, Kim Dalziel, Jon Dorling, Alan Downs, Peter G. Davis, Zoe Daskalopoulou, Amanda Forster, Michaela Graham-Travis, Nigel J. Hall, Marie Hubbard, Madeleine Hurd, Pollyanna Hardy, Rod Hunt, Ann Kennedy, Andrew King, Louise Linsell, Brett J. Manley, David Murray, Tracy K. Mitchell, Heather O’Connor, Shalini Ojha, Charles C. Roehr, Oliver Rivero-Arias, Kayleigh Stanbury, Jacqueline Taylor, Lyvonne Tume, Richard Welsh, Joy Wiles, Kerry Woolfall, Lauren Young, Calum Roberts, Chris Gale, the neoGASTRIC collaborative group, Peter G. Davis, Brett J. Manley, Shalini Ojha, Caz Stokes, The’ Thwin, Amanda McKenna, Sharon Hughes, Lucy Lewis, Nigel Brooke, Sarah Farmer, Laura Dalton, Rachel Wane, Joan Baticula, Abigail Simmons, Heidi Hollands, Jasmine Stares, Nigel Brooke, Sarah Farmer, Geraint Lee, Helen Broomfield, Joanna Robinson, Sally Senior, Jennifer Jones, Oliver Rackham, Matthew Pickup, Catrin Johns, Becky Icke, Amy Hoskin, Ruth Davies, Tracey Benn, Elizabeth Lek, Laura Fowler, Komal Lal, Jiji Ouseph, Philip Amato Gauci, Alys Capell, Helen Wilson, Narendra Aladangady, Paul Fleming, Fiona Stacey, Helen Yates, Leanne Sherris, Tammana Williams, Elizabeth Taylor, Geza Vass, Eleri Adams, Bryony Ward, Juliet Newson, Jennifer Smith, Lindsay Carlton, Nele Legge, Amanda Beesley, Laura Burgess, Joanne Windrow, Doris Iyamabo, Carly Reynolds, Jessica Nutting, Helen Harizaj, Shiranthi Jayasekara, Cynthia Kenala, Amy Nichols, Paul Clarke, Samantha Scott, Lucy Pocock, Jasmine Myhill, Richard Nicholl, Trinity Okome, Sara Jaarour, Saima Ali, Kamaal Mughal, Helen Wilson, Omotakin Omolokun, Mollie Jones, Mark Johnson, Catherine Postlethwaite, Abby Parish, Mollie Jones, Victoria Melvin, Samantha Davies, Tim Scorrer, Alison Le Poidevin, Batia Gourin, Jay Banerjee, Zoe McClure, Melanie Hayman, Richard Lloyd-Nash, Holly Dickson, Christos Zipitis, Emily Chadwick, Bethan Gilbody, Claire Williams, Helen McDevitt, Susanna Sakonidou, Lee Ann Joyce, Dinakar Seshadri, Samantha O’Brien, Charlotte Lea, Ruth Bowen, Melanie Hayman, Coral Smith, Bharathi Rao, Adam Reynolds, Roberta Carlisle, Olwen Fleck, Michelle Horrocks, Sarah McCullough, Chloe Scott, Natalie Morgan, Richa Gupta, Emma Strogen, Jennifer Middleton, Christina Oliver, Ros Knight, Stefan Zalewski, Julie Groombridge, Nicola Booth, Clare Jennings, Ramamoorthy Madhepalli Varadan, Jasmine Stares, Katherine Burke, Deborah Settle, Paula Brock, Victoria Bevan, Rachel Hobbs, Nigel Kennea, Naomi Hayward, Batia Gourin, Jay Banerjee, Zoe McClure, Pamela Cairns, Liam Mahoney, Louise Millett, Sanjay Salgia, Lisa Frankland, Kevin Chuen Poon, Murali Natti, Alison Davies, Brenda Argus, Ruth Cousins, Elizabeth Day, Chidambara Harikumar, Dawn Egginton, Lisa Brown, Lieve Boel, Mallinath Chakraborty, Sian Elliott, Amarpal Bilkhu, Denise Vigni, Nazakat Merchant, Sankara Narayanan, Clare Dawson, Grace Audu, Maria Sevilla, Nischal Rao, Catherine Spelman, Leanne Taylor, Vimal Vasu, Claire Moloney, Michael Stark, Tara Crawford, Rajesh Viswanathan, Aimi Streeter, Nicola Booth, Clare Jennings, Shakir Saeed, Fawaz Daher, Choudhary Maqsood, Hilary Owen

**Affiliations:** 1 National Perinatal Epidemiology Unit Clinical Trials Unit, Nuffield Department of Population Health, University of Oxford, Old Road Campus, Oxford, UK; 2 Department of Paediatrics, Monash Newborn, Monash Children’s Hospital, Monash University, Clayton, Australia; 3 Chelsea and Westminster Hospital, Neonatal Intensive Care Unit, London, UK; 4 Neonatal Medicine, School of Public Health, Faculty of Medicine, Imperial College London, London, UK; 5 Consumer Advisory Group, NHMRC CRE in Newborn Medicine, Murdoch Children’s Research Institute, Parkville, Australia; 6 Health Economics Group, Melbourne School of Population and Global Health, University of Melbourne, Melbourne, Australia; 7 University of Leeds, Leeds, UK; 8 The Royal Women’s Hospital, University of Melbourne, Melbourne, Australia; 9 James Cook University Hospital, Middlesbrough, UK; 10 University Surgery Unit, Faculty of Medicine, University of Southampton, Southampton, UK; 11 University Hospitals of Leicester NHS Trust, Leicester, UK; 12 Department of Paediatrics, Department of Neonatal Medicine, Monash University, Monash Health Cerebral Palsy Alliance, University of Sydney, Sydney, Australia; 13 University of Oxford, Oxford, UK; 14 Department of Obstetrics and Gynaecology, Mercy Perinatal, Mercy Hospital for Women, The University of Melbourne, Melbourne, Australia; 15 Department of Public Health Policy and Systems, University of Liverpool, Liverpool, UK; 16 University of Nottingham, Nottingham, UK; 17 Faculty of Health and Life Sciences, University of Bristol, Bristol, UK; 18 Edge Hill University, Ormskirk, Alder Hey Children’s NHS FT, Liverpool, UK; 19 SSNAP (Support for Sick Newborn and Their Parents), Oxford, UK; 20 The Ritchie Centre, Hudson Institute of Medical Research, Clayton, Australia; 21 School of Public Health and Centre for Paediatrics and Child Health, Faculty of Medicine, Chelsea and Westminster Campus, Imperial College London, London, UK

**Keywords:** Infant, Preterm, Enteral feeding, Gastric residual volume, Necrotising enterocolitis (NEC), Neonatal care, Randomised controlled trial, Feeding intolerance, Neonatal nutrition, Gastric tube feeds, Protocol

## Abstract

**Background:**

Routine measurement of gastric residual volumes involves regularly aspirating the entire stomach contents to assess the volume and colour of the aspirate to inform feeding. This is an established practice in many United Kingdom and Australian neonatal units for preterm infants receiving gastric tube feeds. The rationale is to assess feed tolerance and to predict and potentially prevent necrotising enterocolitis, a serious gut condition. Routine measurement of gastric residual volumes may also be associated with adverse outcomes and harm, including delayed achievement of full enteral feeds and longer neonatal unit stay. Evidence to support the routine measurement of gastric residuals is poor, and previous small trials have not been generalisable to UK or Australian neonatal care.

**Methods:**

The aim of the neoGASTRIC trial is to test whether avoiding routine measurement of gastric residual volumes in preterm infants reduces the time taken for an infant to reach full enteral feeds without increasing necrotising enterocolitis. neoGASTRIC is an individually randomised controlled trial in neonatal units in the UK and Australia. A target of 7040 infants born before 34 weeks’ gestation will be randomly allocated, prior to receiving 24 h of enteral feeds > 15 ml/kg/day, on a 1:1 basis to have no routine gastric residual volumes measured, or to have gastric residual volumes measured routinely. Opt-out consent will be used with parent and staff views explored as part of an embedded process evaluation. The primary superiority outcome is time to reach full milk feeds ≥ 145 ml/kg/day for three consecutive days. Bell’s stage 2 or 3 necrotising enterocolitis following blinded adjudication will be the key secondary, non-inferiority safety outcome. Other neonatal core outcomes and health care resource use and costs prior to discharge will be evaluated.

**Discussion:**

neoGASTRIC will address a research priority that affects more than 20,000 preterm infants in the United Kingdom and Australia annually. Even modest improvements in clinical outcomes and resource use could result in large clinical benefits and savings at a population level.

**Trial registration:**

ISRCTN 16710849. Prospectively registered on 8 February 2023.

**Supplementary Information:**

The online version contains supplementary material available at 10.1186/s13063-025-09403-7.

## Structured summary {1b}

**Table:** 

Primary registry and trial identifying number {4}.	ISRCTN registration number: 16710849 https://www.isrctn.com/ISRCTN16710849
Secondary identifying numbers	n/a
Sources of monetary and material support {7a and b}	National Institute for Health and Care Research (NIHR) Health Technology Assessment Programme (NIHR134216) and National Health and Medical Research Council NHMRC-NIHR Collaborative Research Grant Scheme (NHMRC2014792)
Primary sponsor and contact information {3b}	United Kingdom Sponsor: Ms Becky Ward Research Governance and Integrity Manager Imperial College London becky.ward@imperial.ac.uk Australian delegated sponsorship organisation: Amy Rodriquez School of Clinical Science, Monash University, Department of Paediatrics, Monash Children’s Hospital, Australia Amy.Rodriquez@monash.edu
Role of sponsor and funder {3c}	Imperial College London, as the Sponsor of the neoGASTRIC Trial, holds a central role in ensuring successful execution and oversight of the study. Their responsibilities include taking overall legal responsibility for the management of the research study, ensuring that it is undertaken according to all regulatory requirements, and reviewing all documents prior to ethical submission. Monash University will act as the Australian delegated sponsorship organisation, taking on delegated responsibilities for participating Australian sites. NHMRC-NIHR Collaborative Research Grant Scheme. The funders have no influence on study design, collection, management, analysis and interpretation of data, writing of the report and the decision to submit for publication.
Contact for public queries	neoGASTRIC@npeu.ox.ac.uk neoGASTRIC | NPEU
Contact for scientific queries	Professor Chris Gale: christopher.gale@imperial.ac.uk Associate Professor Calum Roberts: calum.roberts@monash.edu
Public title	The neoGASTRIC Trial
Scientific title {1}	Avoiding routine gastric residual volume measurement in neonatal critical care (The neoGASTRIC trial)
Countries of recruitment	Australia, United Kingdom
Health condition(s) or problem(s) studied	Preterm infants born at less than 34 + 0 gestational weeks + days, admitted to participating neonatal units in the United Kingdom and Australia.
Interventions	Two pathways of care are being compared: 1. No routine measurement of gastric residual volumes 2. Routine, at least 6-hourly, measurement of gastric residual volumes Both pathways represent standard clinical practice in different neonatal units in the United Kingdom and Australia.
Key inclusion and exclusion criteria	Inclusion criteria: • Gestational age at birth less than 34 + 0 (up to and including 33 + 6) gestational weeks + days. • Nasogastric or orogastric tube in place Exclusion criteria: • Infant has received more than 15 ml/kg/day of milk for more than 24 h • Gastrointestinal surgical condition (including suspected necrotising enterocolitis and focal intestinal perforation) prior to randomisation • Major congenital abnormalities • No realistic prospect of survival • A parent has opted out of infant’s participation in neoGASTRIC
Study type	Multi-centre, pragmatic, unblinded, two-arm, parallel group, randomised, controlled trial with a superiority primary outcome and a non-inferiority key secondary safety outcome, using opt-out consent, with an internal pilot (and embedded process evaluation), and an integrated costing study.
Date of first enrolment	15 June 2023
Sample size	Original sample size 7040. Following consultation with trial oversight committees recruitment will continue until the planned end date of 28 February 2026, recognising that this may result in a higher final sample size.
Primary outcome	Time from birth to reach full milk feeds for 3 consecutive days (at least 145 ml/kg/day where this is considered full enteral feeds, or where breastfeeding and any additional milk is considered equivalent to full enteral feeds)
Key secondary outcome	Necrotising enterocolitis, modified Bell’s stage 2 or greater [1], evaluated by Blinded Endpoint Review Committee, up to discharge home or 44 + 0 gestational weeks + days (whichever is sooner)
Ethics review	UK: London – Riverside Research Ethics Committee (REC). Approvals completed 8 February 2023 Australia: Monash Health Human Research Ethics Committee (HREC), approval date 30 March 2023
Individual trial participant data sharing statement	The study team will share a de-identified dataset with outside investigators upon reasonable request. Investigators may be required to provide evidence of research ethics approval (or exemption) and/or complete a data sharing agreement. Data requests should be directed to the corresponding author for review and consideration. Please be aware that exclusive access to the data will be maintained until after publication of the main trial findings. After this, access to anonymised data may be granted following a detailed review process.

### Protocol version {2}

Protocol Version 4.0 26 November 2025

## Introduction

### Background and rationale {9a}

Preterm infants have immature suck and swallow coordination, and so the use of gastric feeding tubes is standard care for infants below approximately 34 gestational weeks [1–3]. The routine measurement of gastric residual volume involves regularly aspirating, via a nasogastric or orogastric tube, the entire stomach contents to assess the volume and colour of the gastric aspirate. This practice is distinct from the aspiration of a small volume of gastric fluid for pH testing to confirm gastric tube position [4].

Routine measurement of gastric residual volumes is established practice in many neonatal units in the UK, Australia and internationally [2]. The rationale underpinning this practice is to guide feeding decisions, to assess ‘feed intolerance’, and potentially predict and prevent NEC [5, 6]. Large-volume, bilious or blood-coloured gastric aspirates—in conjunction with other signs such as abdominal distension and tenderness, shock and respiratory compromise—are frequently observed in infants with necrotising enterocolitis (NEC). Whether regular monitoring of gastric aspirates allows identification and prediction of NEC early enough to modify disease course and outcome is, however, not well-evidenced [5, 6], but nonetheless remains a key driver of routine measurement in neonatal units around the world [7].

Routine measurement of gastric residual volumes may also be associated with adverse outcomes and harm, including delayed achievement of full enteral feeds [8, 9] which increases the risk of associated complications such as late-onset infection [10, 11] longer neonatal unit stay [12, 13] discomfort, which is a key concern to parents [7], and depletion of gastric secretions [14].

Several small, randomised trials have compared routine with no routine measurement of gastric residual volumes [8, 12, 13, 15–17]. Individually, or when their results are pooled, these trials are underpowered to detect a difference in the incidence of NEC; furthermore, findings from these previous trials are not generalisable to UK National Health Service (NHS) or Australian care settings due to differences in the trial populations and settings.

### Explanation for the choice of comparators {9b}

Two pathways of care are being compared:

1. No routine measurement of gastric residual volumes
2. Routine, at least 6-hourly, measurement of gastric residual volumes

Both pathways represent standard clinical practice in different neonatal units in the UK and Australia; they are based upon UK and international practice [2] and a consensus meeting [7] which involved parents, neonatologists, neonatal nurses, dieticians and trial methodologists.

The allocated pathway of care will be followed:

- For as long as routine gastric residual volume measurement is standard local practice,
- Until gastric feeding tubes are no longer required,
- The infant is discharged home, or
- The infant reaches 44 + 0 gestational weeks + days.

#### Objectives {10}

The aim of the neoGASTRIC trial is to determine whether routinely measuring gastric residual volumes in preterm infants born less than 34 weeks’ gestation reduces the time taken for an infant to reach full milk feeds without increasing harms, up until they are discharged home or reach 44 + 0 gestational weeks + days, whichever is sooner.

#### Primary objective

To determine if not routinely measuring gastric residual volumes, compared to routinely (at least 6-hourly) measuring gastric residual volumes, in preterm infants born less than 34 weeks’ gestation reduces the time to achieve full milk feeds (at least 145 ml/kg/day), up until discharge home or 44 + 0 gestational weeks + days, whichever is sooner.

#### Key secondary objective

To determine if not routinely measuring gastric residual volumes is non-inferior to routinely (at least 6-hourly) measuring gastric residual volumes, in preterm infants born less than 34 weeks’ gestation for the key safety outcome of moderate or severe NEC diagnosed up until discharge home or 44 + 0 gestational weeks + days, whichever is sooner.

#### Other secondary objectives

- To evaluate the impact of not routinely measuring gastric residual volumes compared to routine measurement on other clinical outcomes.
- To assess the resource use and costs from an NHS perspective of not routinely measuring gastric residual volumes compared to routine measurement.

## Methods {11}: patient and public involvement, trial design

Healthcare professionals and neonatal patients/parents were involved in developing the design of the trial and the comparator arms [7, 15].

Twenty-four parents with neonatal experience were involved in a consensus process to identify outcomes for neoGASTRIC; this also included healthcare professionals, methodologists and researchers. ‘Time to full feeds’ and the diagnosis of NEC were prioritised as primary outcomes [16].

Parents and patients have been involved in the development of the opt-out approach to consent in neonatal comparative effectiveness research that will be used in neoGASTRIC [18]. Two parents of extremely preterm infants and the national neonatal charity Bliss developed this approach to simplify opt-out consent, which was subsequently piloted in the WHEAT trial and qualitatively evaluated in 11 families who experienced opt-out consent [15, 19]. The opt-out approach to consent was considered feasible and normalised research while preserving parent autonomy. This opt-out approach to consent has been refined based upon this qualitative work.

For the Australian component of the trial, an Australian Parent Advisory Group has been convened, including several parents of extremely preterm infants and an extremely preterm-born adult. They were positive about the benefits of the trial and specifically felt the opt-out consent process was appropriate if the provided information was sufficiently clear.

Parents with neonatal experience will be part of a Parent Advisory Group (PAG). The group will help to inform the parent-facing material such as Parent Information Sheets and posters and will review any updates made during the conduct of the trial. After results are available, the PAG, together with relevant charities such as Bliss and National Maternity Voices, will help with the communication of results to health professionals.

### Trial design {12}

Multi-centre, pragmatic, unblinded, two-arm, parallel group, randomised controlled trial with a superiority primary outcome and a non-inferiority key secondary safety outcome, using opt-out consent, with an internal pilot (and embedded process evaluation), and an integrated costing study.

## Methods: participants, interventions and outcomes

### Trial setting {13, 14b}

Neonatal units caring for preterm infants, including the following levels of neonatal units: Neonatal Intensive Care Units (NICUs), Local Neonatal Units (LNUs) and Special Care Baby Units (SCBUs) in the UK, and tertiary NICUs in Australia. See Additional file 1: Tables 1a and 1b, for the full list of study sites.

### Eligibility criteria for participants {14a}

#### Inclusion criteria

1. Gestational age at birth less than 34 + 0 gestational weeks + days (up to and including 33 + 6 gestational weeks + days)
2. Nasogastric or orogastric tube in place

#### Exclusion criteria

1. Infant has received more than 15 ml/kg/day of milk for more than 24 h
2. Gastrointestinal surgical condition (including suspected NEC and focal intestinal perforation) prior to randomisation
3. Major congenital abnormalities
4. No realistic prospect of survival
5. A parent has opted out of infant’s participation in neoGASTRIC

Infants enrolled in other interventional studies are eligible for participation in the neoGASTRIC trial wherever this is possible, to be confirmed for individual trials.

### Eligibility criteria for sites and those delivering interventions {14a}

National Health Service (NHS) Neonatal Intensive Care Units (NICUs), Local Neonatal Units (LNUs) and Special Care Baby Units (SCBUs) in the UK; tertiary NICUs in Australia.

### Who will take informed consent? {32a}

In UK and Australian units, potential participants meeting the eligibility criteria will be identified by appropriately trained and experienced neonatal doctors and nurses either before or after admission. The neoGASTRIC trial will use an opt-out approach to consent [16]. Parents or carers will be informed about neoGASTRIC through posters, a leaflet and electronic media, provided when their infant is admitted to the neonatal unit and prior to randomisation. After this information is made available, infants meeting the eligibility criteria will automatically be included in the trial. Parents will have the option to opt out prior to randomisation if they do not wish their infant to be included in the trial. They will almost always have at least 24–48 h to do this, and in many cases, they will have substantially longer (considering the time taken to reach feeds greater than 15 ml/kg/day in many neonatal units). They will also be able to withdraw from the study at any point after their infant is randomised. The opt-out nature of neoGASTRIC means that there will not be a signed consent form.

### Additional consent provisions for collection and use of participant data and biological specimens {32b}

Using the same opt-out process, consent will be obtained for linkage to and use of routinely recorded clinical data for trial purposes. No biological specimens are to be collected.

## Intervention and comparator

### Intervention and comparator description {15a}

No routine measurement of gastric residual volumes: Within this pathway of care, gastric residual volumes will not be measured routinely, although a small volume of gastric fluid will be aspirated for pH testing to confirm gastric tube position as per local/national guidance. Feed tolerance will be assessed by monitoring the infant for symptoms such as vomiting, abdominal tenderness, discolouration or distension, bloody stools or clinical deterioration; see Additional file 2: Fig. 1a.

Routine, at least 6-hourly, measurement of gastric residual volumes: Within this pathway of care, gastric residual volumes will be measured at least every 6 h and used to evaluate feed tolerance as specified by existing local practice. Where local practice is not standardised, units can use the neoGASTRIC trial suggested gastric residual assessment guidance; see Additional file 2: Fig. 1b.

### Criteria for discontinuing or modifying allocated intervention/comparator {15b}

Where an infant develops a condition for which gastric residual measurement is clinically indicated—for example, suspected NEC or gastrointestinal obstruction, or following abdominal surgery—such gastric residual measurement is no longer ‘routine’ so should be undertaken as clinically indicated in both trial arms. When gastric residual measurement is no longer clinically indicated, the infant should resume their allocated care pathway if this is considered clinically appropriate.

### Strategies to improve adherence to intervention/comparator {15c}

Adherence to the allocated care pathway will be recorded at sites by recording the number of gastric residual volume measurements per calendar day until an infant achieves the primary outcome (full enteral feeds). Tools to assist adherence to the trial arm, such as cot cards and stickers to attach to gastric tubes, will be provided to sites, and site monitoring reports indicating non-adherence will be provided quarterly to sites for discussion and to inform local practice. Strategies to improve adherence will also be explored in the embedded process evaluation.

### Concomitant care permitted or prohibited during the trial {15d}

Other aspects of nutritional practice will follow the unit’s usual practice. This includes, but is not limited to, timing of commencement of feeds, speed of increase of enteral feeds and choice of milk where mother’s milk is insufficient. There are no contraindicated medications or interventions, and infants will be able to have all medicines normally prescribed for this population during the trial.

### Ancillary and post-trial care {34}

After an infant reaches the primary outcome, full milk feeds for three consecutive days, trial sites will be asked to continue to practise the allocated trial arm. Daily monitoring of adherence to the allocated trial arm will not occur after the primary outcome is reached, but the presence or absence of routine measuring in the previous calendar day will be recorded when an infant develops signs consistent with possible NEC. As both pathways are routinely practised in the UK and Australia, we do not believe there are any additional risks for infants taking part in this trial. Once the infant completes the trial, at discharge home or 44 + 0 gestational weeks + days (whichever is sooner), the care of the infants will revert to local practice.

### Outcomes {16}

**Table:** 

**Primary outcome**	**Primary outcome measure**	**Time point**
Primary outcome (superiority outcome)	Time (in days) from birth to reach full milk feeds for 3 consecutive days (at least 145 ml/kg/day where this is considered full enteral feeds, or where breastfeeding and any additional milk is considered equivalent to full enteral feeds)	Up to discharge home or 44 + 0 gestational weeks + days (whichever is sooner)
**Secondary outcomes**	**Secondary clinical outcome measures**	**Time point(s) of evaluation**
Key secondary outcome (non-inferiority outcome)	NEC: modified Bell’s stage 2 or greater [15], evaluated by Blinded Endpoint Review Committee (BERC)	Up to discharge home or 44 + 0 gestational weeks + days (whichever is sooner)
Other secondary outcomes	• Severe NEC, confirmed at surgery or leading to death, evaluated by BERC	
	• All-cause mortality	
	• Focal intestinal perforation, evaluated by BERC	
	• Gastrointestinal surgery	
	• Late-onset infection (> 72 h after birth): microbiologically confirmed [19, 20] or clinically suspected infection [21] evaluated by BERC	
	• Duration of neonatal unit stay, in days, including all levels of care	
	• Duration of any parenteral nutrition, in days	
	• Duration with a central venous line in situ, in days	
	• Weight for gestational age standard deviation score	
	• Head circumference for gestational age standard deviation score	
	• Duration of invasive ventilation, in days	
	• Chronic lung disease: receiving oxygen or respiratory support at 36 weeks’ corrected gestational age	
	• Retinopathy of prematurity: treated medically or surgically [22]	
	• Brain injury on imaging: intraventricular haemorrhage grade 3 or 4 and/or cystic periventricular leukomalacia [23]	
	• Any vomiting resulting in feeds being withheld	Up to 14 days from randomisation
	• Number of days feeds withheld at least once	Up to 14 days from randomisation
	• Total number of hours feeds withheld	Up to 14 days from randomisation
	• Breastfeeding	At discharge home or 44 + 0 gestational weeks + days (whichever is sooner)
	• Receiving maternal breastmilk	
**Costing study**	• Number of gastric residual volume measurements	Up to 14 days from randomisation
	• Abdominal x-ray investigations^a^	Up to discharge home or 44 + 0 gestational weeks + days (whichever is sooner)
	• Antibiotic use and surgery for NEC or focal intestinal perforation	
	• Healthcare costs^b^	

^a^Data to inform this outcome will be recorded within the pilot study/process evaluation

^b^Will also include costs of key care items listed under secondary outcomes, for example duration of neonatal unit stay by level of care

### Harms {17}

The safety reporting window for this trial will be from randomisation until the end of trial follow-up (discharge home or 44 + 0 gestational weeks + days, whichever is sooner).

Due to the nature of the patient population, neonates in intensive care, a high incidence of adverse events is foreseeable during their routine care and treatment. Consequently, only those adverse events identified as serious will be recorded for the trial.

#### Foreseeable SAEs which do not require expedited reporting

The following events are expected in the population, and information will be collected by recruiting sites during the intervention period as outcomes; therefore, they do not require reporting as SAEs. Data pertaining to these events will be reviewed by the DMC at a frequency to be determined by the DMC (at least annually).

- Death (unless cause not anticipated in this population)
- NEC or gastrointestinal perforation
- Bronchopulmonary dysplasia or chronic lung disease
- Late-onset infection
- Brain injury on imaging: intraventricular haemorrhage grade 3 or 4 and/or cystic periventricular leukomalacia

#### Foreseeable SAEs relating to known complication(s) of prematurity

Any serious event that is deemed by the investigator to be a known complication of prematurity at that gestational age should not be reported as an SAE but should be recorded in the infant’s medical notes, as per usual practice. They do not require reporting by trial centres as SAEs unless considered that they may be causally related to the allocated pathway of care.

#### SAEs which require expedited reporting

Any other SAEs not detailed above are classed as unforeseeable SAEs and must be reported.

#### Reporting procedures for SAEs

SAEs requiring expedited reporting must be reported as soon as possible after the site becomes aware of the event being defined as serious. The relationship of each adverse event to the allocated pathway of care will be determined by a medically qualified individual, and all SAEs labelled possibly, probably or definitely will be considered as related to the allocated pathway of care. An SAE that is deemed to be related to the allocated care pathway will be assessed by the CI or the Australian CI (or other appropriate delegate) to determine whether the event is expected or unexpected in terms of the current known safety profile of the allocated pathway of care. All related and unexpected SAEs will be submitted to the sponsor and the Research Ethics Committee (REC) that gave a favourable opinion of the trial within 15 days of the CI becoming aware of the event and also to the DMC, sponsor and the R&D offices.

### Participant timeline {18}

**Table:** 

	**Period**						
	**Enrolment**		**Post-randomisation**				**Close-out**
**Timepoint**	**After birth**	**After eligibility confirmed**	**Day 1**	**Day 2**	**Day 3**	**etc**	**Discharge from neonatal unit or 44 + 0 gestational weeks + days**
Enrolment							
Eligibility screen	X						
Opt-out consent	X						
Randomisation		X					
Baseline data		X					
Intervention/comparator							
*Routine* or *no routine* measurement of gastric residual volumes			X	X	X	X	
Assessments							
Daily feeding log			X	X	X	X^a^	
Late-onset infection and gut signs						X^b^	
Data at discharge or **44 + 0 gestational weeks + days**							
Clinical data^c^							X^d^

^a^Daily feeding logs until day 14, and then until the infant achieves full enteral feeds (at least 145 ml/kg/day where this is considered full enteral feeds, or where breastfeeding and any additional milk is considered equivalent to full enteral feeds) for 3 consecutive days, or reaches 44 + 0 gestational weeks + days

^b^Each episode of microbiologically confirmed or clinically suspected late-onset invasive infection, or if an infant has received at least 5 days of treatment for gut signs, if they are transferred with gut signs, or if they have died from gut signs, should be reported throughout the treatment period until hospital discharge

^c^Clinical data is collected via the electronic case report form or from routinely recorded clinical data where applicable

^d^If infant is withdrawn from the trial and parent/carer has asked for no further data collection, the transfer/discharge form can be completed at the time of withdrawal for data up until point of withdrawal

### Sample size {19}

The original planned sample size for this trial is 7040 infants (3520 per arm) individually randomised in neonatal units in the UK and Australia. Multiple births will be randomised to the same arm. Following consultation with trial oversight committees recruitment will continue until the planned end date of 28 February 2026, recognising that this may result in a higher final sample size.

#### Time to full enteral feeding

From the National Neonatal Research Database (NNRD), UK population level neonatal data, the overall mean number of days to full enteral feeding in infants born less than 34 weeks of gestation is 9.4 (standard deviation 10.8) days. To detect a 1-day reduction in the time taken to reach full enteral feeding (important clinically and important to parents) with 90% power and a two-sided 5% significance level, a sample size of 5088 infants (2544 per arm) is required (assuming a standard deviation of 11 days). Based on data from other feeding trials [24, 25] we anticipate 30% multiple births and an intracluster correlation coefficient (ICC) for multiple birth sets of 0.3, leading to an inflation of the sample size by 6% to account for the correlation in outcomes within multiple birth sets [24, 25]. A small level of crossover in the ‘no routine measurement’ group is anticipated, with some measuring of gastric residuals occurring outside of the protocol guidelines; to allow for 10% crossover (non-adherence), the sample size has been inflated by 24% to obtain the same level of power [26]. Applying these inflation rates and assuming a 5% attrition rate to discharge, the total number of infants required is 7040 (3520 per arm).

#### Moderate to severe NEC

From the NNRD the incidence of significant NEC (requiring surgery, leading to death or recorded as part of the National Neonatal Audit Programme) is 3%. With a total sample size of 7040 and a control group event rate of 3%, the trial would have 92% power to detect a non-inferiority margin of 1.5% in the treatment risk difference, with a one-sided 2.5% significance level: non-inferiority can be claimed if the upper bound of the confidence interval of the treatment effect does not exceed 1.5%. These figures also allow for 10% crossover and 5% attrition. The inflation factor to account for the correlation in outcomes within multiple birth sets for NEC is 1% assuming a lower ICC of 0.05.

#### Recruitment {20}

The trial is designed to be pragmatic and low burden through simple, streamlined opt-out consent and minimal data collection. Many sites will be involved, including sites not commonly involved in neonatal trials, for example, smaller non-tertiary units. We plan to enrol from all four nations of the UK and in three Australian states.

## Assignment of interventions: randomisation

### Sequence generation who will generate the sequence {21a}

A Senior Trials Programmer at the Clinical Trials Unit (CTU) will write the web-based randomisation program and hold the allocation codes. Randomisation of infants to either the no routine measurement of gastric residual volumes or the at least 6-hourly measurement of gastric residual volumes will use a 1:1 allocation ratio.

### Sequence generation: type of randomisation {21a, 21b}

The randomisation program will use a probabilistic minimisation algorithm. To ensure balance between the randomised groups, minimisation criteria will comprise: randomising hospital, multiple births, and week of gestational age at birth.

Infants that are part of a multiple birth set (twins or higher order multiples) will be randomised as a multiple: they will all be allocated to the same care pathway. This is based upon feedback from parent representatives, parent organisations and research involving parents and ex-preterm twins [27].

### Allocation concealment mechanism {22}

Infants will be randomised using an online secure central randomisation service to ensure allocation concealment.

### Implementation {23}

Staff at recruiting sites will log into the randomisation website using a unique centre login to randomise participants. The system will confirm eligibility, allocate a unique neoGASTRIC Study ID, and then assign the infant to one arm of the trial.

## Assignment of interventions: blinding

### Who will be blinded {24a}

Because it is not possible to mask the different care pathways, parents and clinicians (including treating clinicians and clinicians recording outcomes) will be unblinded to the allocated care pathway.

A Blinded Endpoint Review Committee (BERC) will undertake a blinded assessment of key clinical outcomes—NEC, focal intestinal perforation, late-onset infection and, where appropriate, time to full feeds.

**Table:** 

Individual	Blinding status	Comments
Parents and infant	Not blinded	Not possible due to the nature of intervention. Parents will be informed which arm of the trial they have been randomised to
Principal Investigator (PI) and other site staff	Not blinded	Not possible due to the nature of intervention. Following randomisation, an email will be sent to the PI and/or other site staff (as agreed locally) confirming allocation
Chief Investigator (CI) UK and Australia	Not blinded to individual infants Blinded to all aggregate data	Not possible for individual infants at their site due to the nature of intervention The CIs also act as PIs for their site—see comments for PIs above, The CI will remain blinded to treatment allocation overall. CIs will not be blinded for infants when involved in evaluating a serious adverse event (SAE)
Database programmer	Not blinded	The database programmer will be responsible for the management of the randomisation database and will also have access to unblinded datasets within the trial database
Trial and data management staff	Not blinded to individual infants Blinded to all aggregate data	Trial and data management staff will have access to unblinded individual records within the clinical database as this is an unblinded study
Trial statistician	Not blinded	The trial statistician will draft the statistical analysis plan before they receive the first unblinded dataset. Thereafter, on request, the trial statistician will have access to the unblinded dataset as this is an unblinded study
Members of the BERC	Blinded	Members of the BERC will assess the relevant Case Report Forms (CRFs) and (if necessary) anonymised medical notes

### How will blinding be achieved {24b]

N/A—this is not a blinded trial.

### Procedure for unblinding if needed {24c}

N/A—this is not a blinded trial.

### Data collection and management

#### Plans for assessment and collection of outcomes {25a}

The majority of trial data will be collected using electronic CRFs and entered directly into the secure OpenClinica Clinical Database Management System (CDMS). The individual participant data will be identified by a study participant number only.

Necrotising enterocolitis, intestinal perforation and neonatal infection will also be assessed by a BERC. BERC reviewers will be medical professionals who are experts in the conditions for which endpoint data is being collected for analysis. See Additional file 4 for more details on the BERC review charter.

### Plans to promote participant retention and complete follow-up {25b}

The Project Management Group (PMG) will remotely monitor the completeness of study documentation and maintain regular contact with all sites to ensure the accuracy and completeness of all study documentation during the infants’ participation in the study. For infants who discontinue participation or where there is a deviation from protocol after the point of randomisation, sites will be asked to record the reasons for this and to continue data collection, where possible, so they can be included in the analysis of outcomes, according to the intention to treat analysis principle.

To support participant retention, several strategies will be implemented throughout the trial. All recruiting sites will receive training at setup, with ongoing support provided via the study website and through direct contact with the trial coordinating team as needed. The Parent Information Sheet (PIS) will be translated into seventeen languages other than English to help make the trial information accessible to families from diverse backgrounds. If an infant is discontinued from the allocated trial pathway but remains in the trial, data collection will continue. If an infant withdraws from the trial completely, data collected up to the point of withdrawal will be used for analysis. Monthly reports on missing data and outstanding CRFs will be shared with sites.

Communication between recruiting sites and Continuing Care Sites (CCSs) will be actively facilitated by the trial coordinating team at the National Perinatal Epidemiology Unit (NPEU) CTU, to support continued data collection following any infant transfers, including to non-CCSs. To maintain engagement, the trial will include structured retention and communication strategies, including staff recognition and family-facing materials. Monthly informal meetings with the coordinating team will provide sites with an opportunity to ask questions, exchange ideas, share key learnings and troubleshoot issues collaboratively.

### Data management {26}

Trial data will be recorded primarily through CRFs using the OpenClinica system from source documents.

OpenClinica is a CDMS used by the NPEU CTU to undertake the management, storing and curation of clinical data. The application is hosted in the UK by an ISO 27001:2013 (Information Security) accredited third party. Updates or new versions of the application are implemented by OpenClinica.

CRF entries will be considered source data if the CRF is the site of the original recording. Clinical data will be entered into a validated database, with validation checks for accuracy. Participant names and identifiable information will be stored in a separate administrative database. Electronic and paper records will be stored securely, with restricted access to authorised personnel. Electronic files will be stored on a secure server, with regular backups and restricted entry. Archiving will follow local guidelines for at least 25 years in the UK and in Australia until the youngest participant reaches the age of 33, with a review of further archiving needs based on data protection laws.

At the end of the trial, all participant data will be transferred to Imperial College London for long-term follow-up, in compliance with data sharing agreements. Trial data for Australian infants only will be transferred securely to Monash University in Australia.

The detailed Data Flow is available in Additional file 3.

### Confidentiality {33}

The trial will adhere to strict data privacy regulations, including the General Data Protection Regulation (GDPR) and Data Protection Act 2018. All documents will be stored securely at the NPEU CTU and accessible only to authorised trial staff. Personal identifiers and trial data will be stored in a separate database linked by the infant’s trial number. After the trial is completed and reports are published, data will be archived in a secure location with controlled access.

### Process evaluation sub-study {12, 20, 27d}

#### Design

The trial includes a mixed methods process evaluation to evaluate the UK pilot phase trial processes, to inform the successful conduct of the ongoing neoGASTRIC trial. Involving site observations, surveys, interviews and focus groups with parents of eligible infants and site staff, the objectives are to review: (1) gastric residual volume measurement processes and protocol adherence, (2) acceptability of not routinely measuring gastric residual volumes, (3) experience of recruitment and opt-out consent, clinical equipoise and (4) staff training needs to inform ongoing trial conduct and training. Topic guides and questionnaires were developed using trial feasibility findings [28].

#### Recruitment and sampling

The first four UK sites open to recruitment will be invited to take part in the process evaluation. Staff will invite parents of the first 20 eligible infants, including those who ‘opt out’ to complete a questionnaire before leaving hospital and/or to register interest in participating in an interview with a University of Liverpool researcher within a month. Staff will be invited to complete a questionnaire relating to recruitment and trial processes for the same 20 infants and take part in an online focus group after 4–6 months of trial recruitment. Site observations will take place at participating sites during the pilot phase.

Based on previous studies, we anticipate interviewing 15–25 parents and conducting 2–4 focus groups to reach information power [29, 30]. We anticipate around 80 parents and staff questionnaires related to 20 infants per site.

#### Analysis

Process evaluation data analysis will be informed by Normalization Process Theory [30]. Digital audio recordings will be transcribed verbatim by a transcription company (UK Transcription, Brighton, UK), checked and anonymised before being imported into NVivo V.14 software for organising and coding. Reflexive thematic analysis will be used for qualitative data, whilst questionnaire data will be inputted into Statistical Product and Service Solutions (SPSS), and descriptive statistics will be used.

## Statistical methods

### Statistical methods for primary and secondary outcomes {27a}

Statistical analyses will be conducted according to a pre-specified statistical analysis plan (SAP), which will be available in a separate document. The SAP will provide detailed information on the methods used to analyse the primary and secondary outcomes, including any adjustments for covariates.

The *routine at least 6-hourly measurement of gastric residual volumes* group will be used as the reference group in all analyses.

#### Primary outcome

The mean, standard deviation, median and interquartile range will be presented by randomised group. For the comparative analysis, a mixed linear regression will be fitted, with mean differences and 95% confidence intervals presented where model assumptions are satisfied. If normality assumptions are not met, quantile regression models will be used with median differences and 95% confidence intervals presented. The model will be adjusted for minimisation factors, which include recruiting centre, gestational age at birth, and whether the infant is one of a multiple birth. Centre will be treated as a random effect, while all other factors will be treated as fixed effects. Correlation between recruited siblings from multiple births will be accounted for by nesting the ‘multiple’ cluster within centre, where technically possible. Both crude and adjusted effect estimates will be presented, but the primary inference will be based on the adjusted estimates.

#### Secondary outcomes

For binary outcomes, including the key secondary outcome of NEC, numbers and percentages will be provided. Risk ratios and 95% confidence intervals will be calculated using a mixed binomial, or Poisson model with a log link and robust variance estimator should the binomial model fail to converge. Risk differences will also be calculated using a mixed binomial model or Poisson model with an identity link. For continuous outcomes, mean and standard deviations or median and interquartile ranges will be presented. Mixed linear regression models will be applied, with mean differences and 95% confidence intervals presented, where model assumptions are satisfied. Skewed continuous outcomes will be analysed using quantile regression models, with median differences and 95% confidence intervals presented.

Secondary outcomes that are not tested will be presented using summary statistics. No tests of statistical significance will be performed, and no confidence intervals will be calculated between the randomised groups for the untested secondary outcomes.

### Who will be included in each analysis {27b}

#### Primary outcome

The primary analysis for the primary outcome of time to full feeds will be based on a modified intention-to-treat (ITT) approach; infants with outcome data available will be analysed in the groups to which they are assigned, regardless of deviation from the protocol or procedure received.

#### Secondary outcomes

For the key secondary outcome (non-inferiority outcome), the primary analysis will be based on a per-protocol population; infants with outcome data available, excluding those considered non-adherent before reaching full feeds, will be analysed in the groups to which they are assigned.

Non-adherence to trial intervention will be defined as the following occurring on two or more consecutive calendar days:

Babies allocated to the *No routine measurement of gastric residual* *volumes* arm: Two or more gastric residual volume measurements. Where gastric residual volume measurements are undertaken during assessment for a clinical indication or concern, this will not be counted as non-adherence.

Babies allocated to the *Routine measurement of gastric residual volumes* arm: Less than three gastric residual volume measurements.

All other secondary outcomes will also be analysed using a modified ITT approach.

### How missing data will be included in each analysis {27c}

Missing data will be described, for example, by presenting the number of individuals in the missing category. Missing data as a result of infants being lost to follow-up is expected to be minimal for short-term outcomes. Infants who are missing their primary outcome data will be considered lost to follow-up. The number and percentage of infants lost to follow-up will be reported with the reasons recorded. Baseline characteristics and some secondary outcome data will also be available from the NNRD for all UK-based participants. This data will be used to impute any missing values for variables where it is available. A sensitivity analysis will be carried out where any data that has been imputed from the NNRD is excluded.

### Methods for additional analyses (e.g., subgroup analyses) {27d}

#### Subgroup analyses

The consistency of the treatment effect on the time to full feeds and NEC by gestational age group, birth weight < 10th centile for gestational age, sex and country will be assessed using the statistical test of interaction, with the associated *p*-value, subgroup-specific treatment effects and 95% confidence intervals presented.

#### Secondary analyses

A per-protocol analysis for the primary outcome and a modified ITT analysis for the key secondary outcome will be conducted applying the same statistical models as used for the primary analyses of these outcomes.

#### Costing study

An integrated analysis will assess the resource use and costs associated with routine measurement and with no routine measurement of gastric residual volumes.

The analysis, conducted from a healthcare system perspective, will utilise routinely available data and hospital records review for individual infants at discharge home or 44 + 0 gestational weeks + days. Key healthcare resource use captured will include the number of gastric residual measurements taken and abdominal x-rays performed (based upon the observation phase of the process evaluation), treatment for NEC and infections, parenteral nutrition, duration of neonatal unit stay at intensive care, high dependency care and special care levels, and hospital transfers.

Regardless of whether routine measurement of gastric residual volumes could bring additional benefit or be stopped without causing harm, an accurate estimate of the resources required by this activity will be essential to inform healthcare budgeting. A micro-costing analysis of gastric residual volume measurements will be carried out in a selected number of infants and sites in the UK to understand this. A data collection form will be developed with clinical input to capture the key resource use involved in this activity from the start of the process until a decision to administer or withhold feeds is made. The questionnaire will collect information about who performs the initial aspiration and whether additional staff support is required, equipment/disposable items involved and pH measurements results. For the UK sites, gastric residual volume measurements will be observed for a representative sample of infants across 4 centres involved in the process evaluation sub-study. The resulting resource use observations will be costed and used to calculate a mean cost per gastric residual volume measurement, which will then be used to cost each measurement recorded for each infant in the trial. For the Australian sites, pragmatic adjustments may be made based on local clinical practice.

The main source of resource use data associated with neonatal care will be the NNRD in the UK and trial CRF and hospital administrative records in Australia. Resource use will be costed using unit costs from established national sources in both countries. In the UK, this will include the NHS National Cost Collection [31], the Unit Costs of Health and Social Care [32], and the NHS Electronic Drug Tariff [33]. In Australia this includes the Enterprise Agreement for medical and nursing staff, National Hospital Cost Data Collection and Medicare Australia. Use and costs for each category of resources, as well as total costs, will be summarised using means and standard deviations. Comparisons between trial arms will be via mean differences and 95% confidence intervals. These data, reported alongside the trial’s primary and secondary clinical outcomes in the form of a cost consequence analysis, will form the basis of a health economics study published separately from the principal trial manuscript. Sensitivity analyses will assess the impact of key uncertainties in the analysis upon the base-case results. All analyses will be conducted in line with good practice guidance for health economic analyses [33].

### Interim analyses {28b}

The Data Monitoring Committee (DMC), independent of the applicants and Trial Steering Committee (TSC), will review the progress and accumulating data from the trial at least annually and provide advice on the conduct of the trial to the TSC. DMC meetings will include open and closed sessions. The open session will cover recruitment, data quality and pooled safety data. The closed session will focus on accumulating efficacy and safety data by trial arm. Outcomes will be reported with summary statistics, but treatment effects, confidence intervals and *p*-values will be excluded unless requested by the DMC.

### Protocol and statistical analysis plan {5}

The trial protocol is available on the ISRCTN registry: 10.1186/ISRCTN16710849.

The statistical analysis plan will be finalised prior to the end of participant follow-up and made available on the registry page.

## Oversight and monitoring

### Composition of the coordinating centre and trial steering committee {29}

The trial will be led by the NPEU CTU. The NPEU CTU will support trial coordination, data management, quality assurance, statistical and health economic analysis and dissemination. Monash University will lead the coordination of the Australian arm of the trial. The process evaluation will be led by the University of Liverpool.

The trial will be supervised by the PMG, which will comprise the CIs, NPEU CTU staff, Australian Trial Coordinator and members of the University of Liverpool process evaluation team. The PMG will report to the TSC, which is accountable to the Trial Sponsor. The trial will be overseen by a TSC consisting of an independent chair and at least two other independent members. The CI and CTU Director will also sit on the TSC. The TSC Charter, outlining roles, responsibilities and conduct, will be established at the initial meeting.

### Project Management Group (PMG)

- Roles and responsibilities: The trial will be supervised on a day-to-day basis by the project management team.
- Relationship to trial sponsor and funders: This group reports to the trial steering committee, which is responsible to the trial funders.
- Outline of membership: CI, ACI, NPEU CTU staff including: CTU Director, Head of Operations, Senior Trials Manager, Head of Trials Programming, Trial Statistician, and Australian Trial Coordinator.

### Trial Steering Committee (TSC)

- Roles and responsibilities: The role of the TSC is to provide overall supervision for a trial on behalf of the Trial Sponsor and Trial Funder and to ensure that the trial is conducted to the rigorous standards set out in the Department of Health’s Research Governance Framework for Health and Social Care and the Guidelines for Good Clinical Practice.
- Relationship to trial Sponsor and funders: The TSC acts as the oversight body for this trial on behalf of the NIHR Health Technology Assessment (HTA), NHMRC and sponsor.
- Outline of membership: The majority of members of the TSC, including the Chair and Vice-Chair, are independent of the trial. Non-independent members will also form part of the TSC.
- Names of chair and members:
  o Chair: Professor Andy Ewer
  o Independent members: Professor Eleanor Molloy, Ms. Kelly Bailey, Dr. Anne Beissel, Ms. Bryley Conley, Dr. Catherine Harrison, Professor Graeme MacLennan, Professor Sanjay Patole, and Associate Professor Lynn Sinclair
  o Non-independent members: Professor Chris Gale, Associate Professor Calum Roberts, and Associate Professor Pollyanna Hardy

### Blinded Endpoint Review Committee (BERC)

- Roles and responsibilities: To review cases of microbiologically confirmed or clinically suspected late-onset infection, spontaneous intestinal perforation and NEC, determining if they fulfil the trial protocol definition in order to harmonise and standardise assessments. The committee is intended to enhance the consistency, validity and integrity of the trial’s endpoints and/or outcomes.
- Relationship to trial sponsor and funders: All members of the BERC are co-applicants on the trial.
- Outline of membership: BERC reviewers will be professionals who are experts in the conditions for which endpoint data is being collected for analysis. Reviewers are not required to be independent of the trial, as reviews will be carried out blinded to allocation; the potential for bias in the absence of knowledge of the allocation is considered minimal. Reviewers will not review infants who received care at their hospital. The reviewers will be deemed to have no financial conflicts of interest other than being trial co-applicants.

### Composition of the data monitoring committee, its role and reporting structure {28a}

The DMC will consist of three independent members, including a chair, clinician, and statistician, who are separate from the trial team and TSC. During recruitment, the DMC will meet annually or more frequently as needed to review trial conduct, progress, and accumulating data. The DMC will make recommendations to the TSC. The DMC Charter, outlining roles, responsibilities and conduct, will be established at the initial meeting.

#### Data Monitoring Committee (DMC)

- Roles and responsibilities: The role of the DMC is to safeguard the interests of trial participants, potential participants, their families, their carers, investigators, and the sponsor; to assess the safety and efficacy of the intervention during the trial, and monitor the trial’s overall conduct, and protect its validity and credibility.
- Relationship to trial sponsor and funders: The DMC provides advice to the TSC, who will then inform the Sponsor and Funder.
- Outline of membership: The members of the DMC are independent of the trial (e.g., are not involved with the trial in any other way and will not have competing interests that could impact on the trial). They are nominated by the investigators and approved by the NIHR HTA.
- Names of chairs and members:
  o Chair: Dr. David Gillespie
  o Independent members: Dr. Ann Hickey and Professor Jos Latour

### Frequency and plans for auditing trial conduct {29}

The Principal Investigator (PI) will oversee the trial at their site, including recruitment, staff training, and ensuring data quality.

The NPEU CTU will develop a central monitoring plan based on the risk assessment. Recruitment patterns and data will be closely monitored. Any unexpected findings or outliers will be investigated, potentially leading to targeted site monitoring. Routine monitoring or auditing will not be conducted unless necessary, as determined by central monitoring.

### Protocol amendments {31}

The NPEU CTU will submit and, where necessary, obtain approval from the Sponsor, REC (Research Ethics Committee) and HRA (Health Research Authority) for all substantial amendments to the approved documents. Monash University will submit and obtain approval from the relevant Australian Human Research Ethics Committee for all such amendments. Trial sites and recruiting teams will be notified of all substantial amendments as these arise. Trial participants will be recruited using up-to-date documents; participants who have finished trial participation will not be informed of protocol amendments.

### Dissemination policy {8}

Full details of the trial will be made available to parents of infants enrolled in the trial via the trial website: https://www.npeu.ox.ac.uk/neogastric. Information will also be disseminated through charities such as Bliss, Maternity Voices and Supporting Sick Newborn And their Parents (SSNAP) in the UK and Miracle Babies Foundation and Life’s Little Treasures in Australia.

The final research findings will be disseminated by conventional academic outputs, including publication in high-impact journals and presentations at prominent national and international conferences. A key part of planned dissemination will be through updated guidelines, including British Association of Perinatal Medicine (BAPM) and National Institute for Health and Care Excellence (NICE) guidelines in the UK, and at individual unit level and state/territory level guidance within Australia. As nurses will be key to the implementation of any results, further dissemination will be aimed at nurse education forums and resources. Infographics and standardised presentations will be prepared for incorporation and delivery in national or state level neonatal nursing program curriculums.

## Discussion

Over 20,000 infants are born annually before 34 gestational weeks in the UK and Australia, and many millions more internationally. These infants require gastric tube feeds every few hours because their suck and swallow reflexes are immature. It is common practice to have the volume and appearance of the stomach contents—gastric residual volumes—measured prior to gastric feeds. Although this practice is not evidence based and may be harmful [34], it is deeply ingrained in neonatal practice in the UK [35] and internationally—in part due to a belief that gastric residual volumes can predict and prevent NEC [36].

Small single centre trials from India [23] and the United States of America [4] have shown that stopping routine measurement of gastric residual volumes leads to faster establishment of full feeds. However, these studies have not led to changes in the UK or international practice, because they have been unable to examine the impact on NEC [1], and were conducted in settings that are not generalisable to current neonatal care as delivered in the UK and Australia.

The neoGASTRIC trial seeks to determine whether no routine measurement of gastric residual volumes results in a shorter time taken to establish full milk feeds without increasing harms such as NEC. To detect a 1-day reduction in the time taken to reach full enteral feeding (important clinically and important to parents) and as NEC is rare in infants born before 34 weeks’ gestation, this trial needs to be larger than any previous individually randomised trial of preterm infants, with a planned sample size of 7040 infants. Optimal feeding for preterm infants was identified as a research priority by the James Lind Alliance [37], and specifically as a priority in relation to this research question by parents and patients [3].

To achieve the planned sample size within 3 years of recruitment, we have designed the neoGASTRIC trial to be pragmatic, simple and low burden for parents and families and for neonatal clinical teams. It will use a simple, streamlined approach to consent that has been developed with parents and families of infants that received neonatal care and that uses an opt-out approach to consent. A nested process evaluation will examine the views of parents and healthcare professionals of this approach to consent.

If we can demonstrate that not routinely measuring gastric residual volumes is beneficial and safe for preterm infants, this will improve their care internationally and reduce unnecessary resource use. Conversely, if we show that routine measurement of gastric residual volumes is beneficial in reducing NEC, this trial will identify a simple preventative approach to help prevent a feared neonatal condition.

## Trial status

Protocol Version 4.0, dated 26 November 2025. neoGASTRIC participant recruitment is still underway. Recruitment began in June 2023 and will continue until February 28 2026.

## Supplementary Information

Additional file 1. List of Study Sites.

Additional file 2. Intervention.

Additional file 3. Data flow.

Additional file 4. BERC charter.

Additional file 5. SPIRIT checklist.

## Data Availability

Data requests should be directed to the corresponding author for review and consideration. Please note that exclusive access to the data will be maintained until the publication of the main study findings. Subsequently, access to anonymised data may be granted upon a detailed review process.

## Abbreviations

ACI: Australian Chief Investigator
AE: Adverse event
BAPM: British Association of Perinatal Medicine
BERC: Blinded Endpoint Review Committee
CDMS: Clinical Database Management System
CCS: Continuing Care Site
CI: Chief Investigator
CRF: Case report form
CTU: Clinical Trials Unit
DMC: Data Monitoring Committee
GDPR: General Data Protection Regulation
HRA: Health Research Authority
HREC: Human Research Ethics Committee
HTA: Health Technology Assessment
ICC: Intracluster Correlation Coefficient
ISRCTN: International Standard Randomised Controlled Trial Number
ITT: Intention-to-treat
LNU: Local Neonatal Unit
NEC: Necrotising Enterocolitis
NHMRC: National Health and Medical Research Council
NHS: National Health Service
NICE: National Institute for Health and Care Excellence
NICU: Neonatal Intensive Care Unit
NIHR: National Institute for Health and Care Research
NNRD: National Neonatal Research Database
NPEU: National Perinatal Epidemiology Unit
PAG: Parent Advisory Group
PI: Principal Investigator
PIS: Parent Information Sheet
PMG: Project Management Group
R&D: Research & Development
REC: Research Ethics Committee
SAE: Serious adverse event
SAP: Statistical analysis plan
SCBU: Special Care Baby Unit
SPSS: Statistical Product and Service Solutions
SSNAP: Support for Sick Newborns and their Parents
TSC: Trial Steering Committee
UK: United Kingdom
