# Supplementary material for: Avoiding routine gastric residual volume measurement in neonatal critical care (the neoGASTRIC trial): study protocol for a multi-centre, unblinded, randomised, controlled trial
Source: Trials. 2026 Jan 8;27:106. doi: 10.1186/s13063-025-09403-7 (PMC12874682; doi:10.1186/s13063-025-09403-7)
Supplement: Supplementary file 1 — Additional file 1. List of Study Sites. [file 13063_2025_9403_MOESM1_ESM.pdf]

Additional file 1 - List of Study Sites

Table 1a - neoGASTRIC recruiting sites

NO.	SITE NAME	AREA	COUNTRY
**1**	Arrowe Park Hospital	Wirral	UK
**2**	Bassetlaw District General Hospital	Worksop	UK
**3**	Bradford Royal Infirmary	Bradford	UK
**4**	Chelsea & Westminster Hospital	London	UK
**5**	Derriford Hospital	Plymouth	UK
**6**	Diana, Princess of Wales Hospital	Grimsby	UK
**7**	Doncaster Royal Infirmary	Doncaster	UK
**8**	Evelina London Children's Hospital	London	UK
**9**	Glan Clwyd District General Hospital	Rhyl, Denbighshire	UK
**10**	Glangwili General Hospital	Carmarthen	UK
**11**	Great Western Hospital	Swindon	UK
**12**	Hillingdon Hospital	Uxbridge, London	UK
**13**	Hinchingbrooke Hospital	Huntingdon	UK
**14**	Homerton Hospital	London	UK
**15**	Hull Royal Infirmary	Hull	UK
**16**	James Cook University Hospital	Middlesbrough	UK
**17**	Jessop Wing	Sheffield	UK
**18**	John Radcliffe Hospital	Oxford	UK
**19**	Leicester Royal Infirmary	Leicester	UK
**20**	Liverpool Hospital	Liverpool, NSW	AUS
**21**	Liverpool Women's Hospital	Liverpool	UK
**22**	Luton & Dunstable University Hospital	Luton	UK
**23**	Medway Maritime Hospital	Gillingham, Kent	UK
**24**	Monash Children's Hospital	Melbourne, Victoria	AUS
**25**	Norfolk and Norwich University Hospital	Norwich	UK
**26**	Northwick Park Hospital	Harrow, London	UK

**27**	Peterborough City Hospital	Peterborough	UK
**28**	Prince Charles Hospital	Merthyr Tydfil	UK
**29**	Princess Anne Hospital	Southampton	UK
**30**	Princess of Wales Hospital	Bridgend	UK
**31**	Princess Royal Hospital	Haywards Heath	UK
**32**	Queen Alexandra Hospital	Portsmouth	UK
**33**	Queen Charlotte's & Chelsea Hospital	London	UK
**34**	Queen's Hospital	Romford, London	UK
**35**	Royal Alexandra Hospital	Paisley	UK
**36**	Royal Bolton Hospital	Bolton	UK
**37**	Royal Cornwall Hospital (Treliske)	Truro	UK
**38**	Royal Derby Hospital	Derby	UK
**39**	Royal Devon and Exeter Hospital*	Exeter	UK
**40**	Royal Jubilee Maternity Hospital	Belfast	UK
**41**	Royal Oldham Hospital	Oldham	UK
**42**	Royal Preston Hospital	Preston	UK
**43**	Royal Prince Alfred Hospital	Sydney, NSW	AUS
**44**	Royal United Hospital	Bath	UK
**45**	Royal Victoria Infirmary	Newcastle upon Tyne	UK
**46**	Saint Mary's Hospital	Manchester	UK
**47**	Scunthorpe General Hospital	Scunthorpe	UK
**48**	Singleton Hospital	Swansea	UK
**49**	Southmead Hospital	Bristol	UK
**50**	St George's Hospital	London	UK
**51**	St Mary's Hospital	London	UK
**52**	St Michael's Hospital	Bristol	UK
**53**	Stoke Mandeville Hospital	Aylesbury	UK
**54**	The Grange University Hospital	Cwmbran	UK
**55**	The Royal Women's Hospital	Melbourne, Victoria	AUS
**56**	Tunbridge Wells Hospital	Tunbridge Wells	UK

**57**	University Hospital of North Tees	Stockton-on-Tees	UK
**58**	University Hospital of Wales	Cardiff	UK
**59**	University Hospital Wishaw	Wishaw	UK
**60**	Watford General Hospital	Watford	UK
**61**	West Middlesex University Hospital	Isleworth, London	UK
**62**	Whittington Hospital	London	UK
**63**	William Harvey Hospital	Ashford, Kent	UK
**64**	Women's and Children's Hospital	Adelaide, South Australia	AUS
**65**	Wrexham Maelor Hospital	Wrexham	UK
**66**	Wythenshawe Hospital	Manchester	UK
**67**	Ysbyty Gwynedd District General Hospital	Bangor	UK

*

Royal Devon and Exeter Hospital was a recruiting site and, after achieving their recruitment target, became a Continuing Care Site (CCS).

Table 1b - neoGASTRIC Continuing Care Sites

**NO.**	**HOSPITAL NAME**	**AREA**	**COUNTRY**
**1**	Antrim Area Hospital	Antrim	UK
**2**	Barnet Hospital	Barnet	UK
**3**	Calderdale Royal Hospital	Halifax	UK
**4**	Chesterfield Royal Hospital	Chesterfield	UK
**5**	Conquest Hospital	St Leonards on Sea	UK
**6**	Countess of Chester Hospital	Chester	UK
**7**	Darent Valley Hospital	Dartford	UK
**8**	Darlington Memorial Hospital	Darlington	UK
**9**	East Surrey Hospital	Redhill	UK
**10**	Epsom General Hospital	Epsom	UK
**11**	George Eliot Hospital	Nuneaton	UK
**12**	James Paget Hospital	Great Yarmouth	UK
**13**	Kettering General Hospital	Kettering	UK
**14**	King's Mill Hospital	Sutton-in-Ashfield	UK

**15**	Kingston Hospital	Kingston upon Thames	UK
**16**	Lincoln County Hospital	Lincoln	UK
**17**	Macclesfield District General Hospital	Macclesfield	UK
**18**	Milton Keynes University Hospital	Milton Keynes	UK
**19**	North Tyneside General Hospital	North Shields	UK
**20**	Pilgrim Hospital	Boston	UK
**21**	Pinderfields General Hospital	Wakefield	UK
**22**	Poole Hospital	Poole	UK
**23**	Queen Elizabeth Hospital	Woolwich	UK
**24**	Rotherham Hospital	Rotherham	UK
**25**	Royal Berkshire Hospital	Reading	UK
**26**	Royal Free Hospital	London	UK
**27**	Russells Hall Hospital	Dudley	UK
**28**	St Helens Hospital	St Helens	UK
**29**	St Helier Hospital	Carshalton	UK
**30**	St Mary's Hospital	Isle of Wight	UK
**31**	Stepping Hill Hospital	Stockport	UK
**32**	Sunderland Royal Hospital	Sunderland	UK
**33**	Tameside General Hospital	Ashton-under-Lyne	UK
**34**	The Ulster Hospital	Belfast	UK
**35**	University Hospital Lewisham	Lewisham	UK
**36**	University Hospital of North Durham	Durham	UK
**37**	Warwick Hospital	Warwick	UK
**38**	Whiston Hospital	Whiston	UK
