# Supplementary material for: Avoiding routine gastric residual volume measurement in neonatal critical care (the neoGASTRIC trial): study protocol for a multi-centre, unblinded, randomised, controlled trial
Source: Trials. 2026 Jan 8;27:106. doi: 10.1186/s13063-025-09403-7 (PMC12874682; doi:10.1186/s13063-025-09403-7)
Supplement: Supplementary file 2 — Additional file 2. Intervention. [file 13063_2025_9403_MOESM2_ESM.pdf]

Additional File 2 - Intervention Description Figures

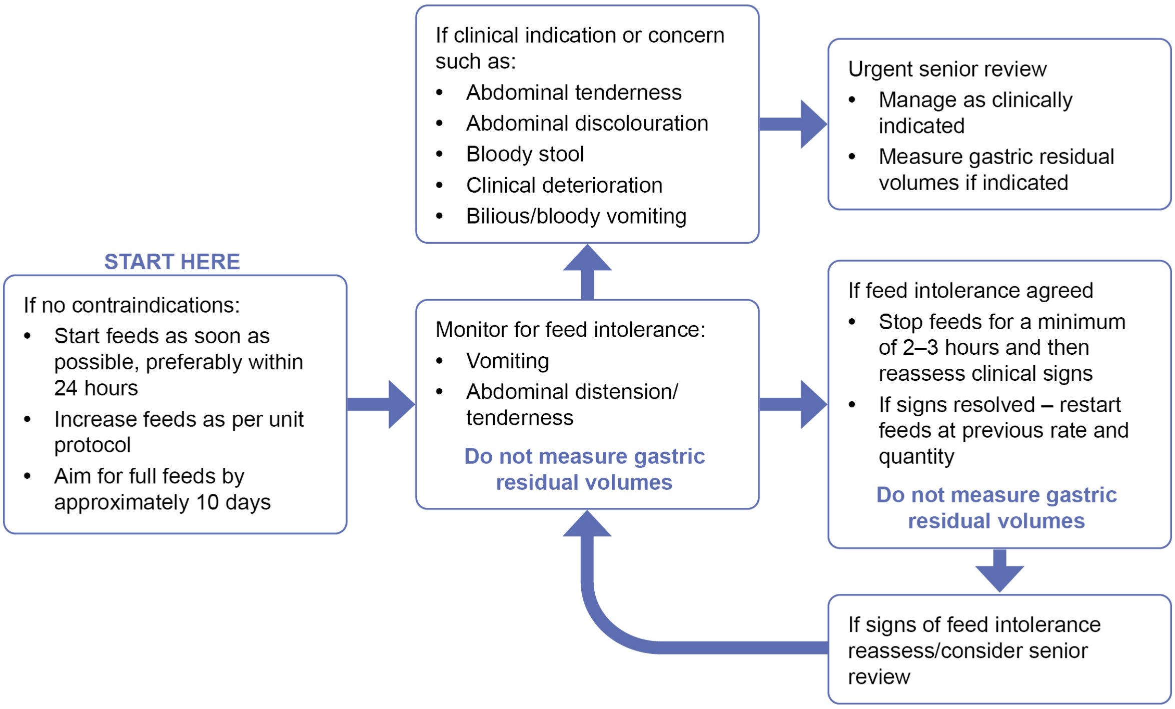

Figure 1a - Suggested management within the No routine measurement of gastric residual volumes pathway

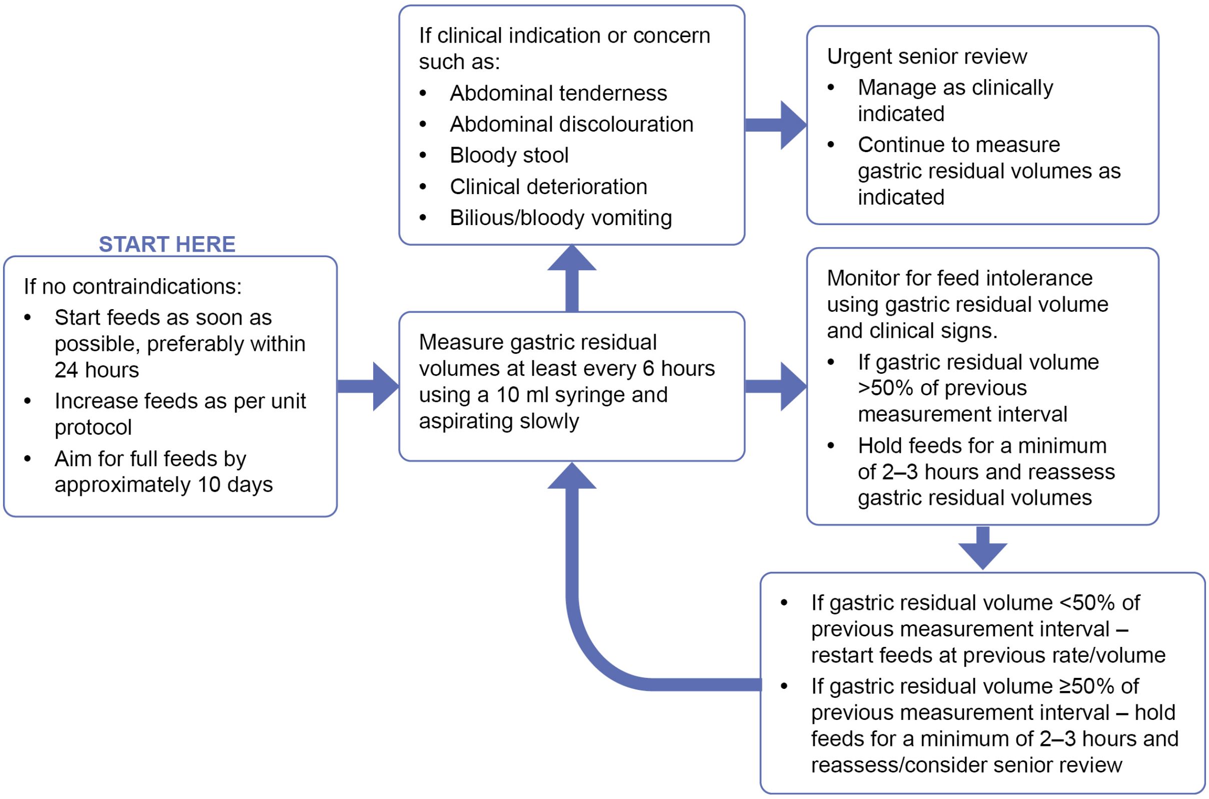

Figure 1b - Suggested management within the 'Routine, up to 6 hourly, measurement of gastric residual volumes' pathway
