# Supplementary material for: Avoiding routine gastric residual volume measurement in neonatal critical care (the neoGASTRIC trial): study protocol for a multi-centre, unblinded, randomised, controlled trial
Source: Trials. 2026 Jan 8;27:106. doi: 10.1186/s13063-025-09403-7 (PMC12874682; doi:10.1186/s13063-025-09403-7)
Supplement: Supplementary file 3 — Additional file 3. Data flow. [file 13063_2025_9403_MOESM3_ESM.pdf]

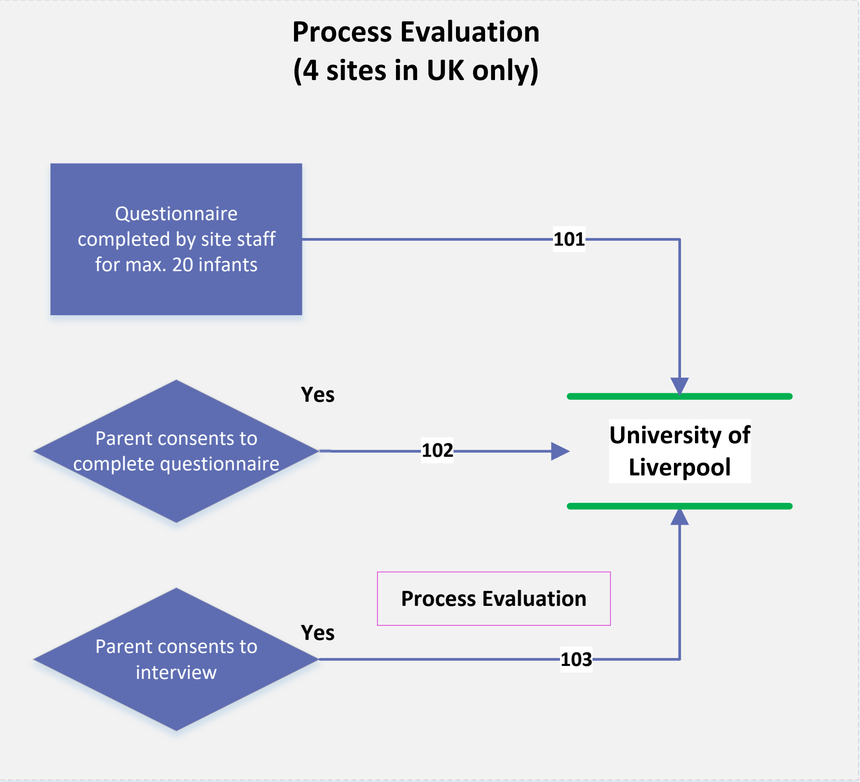

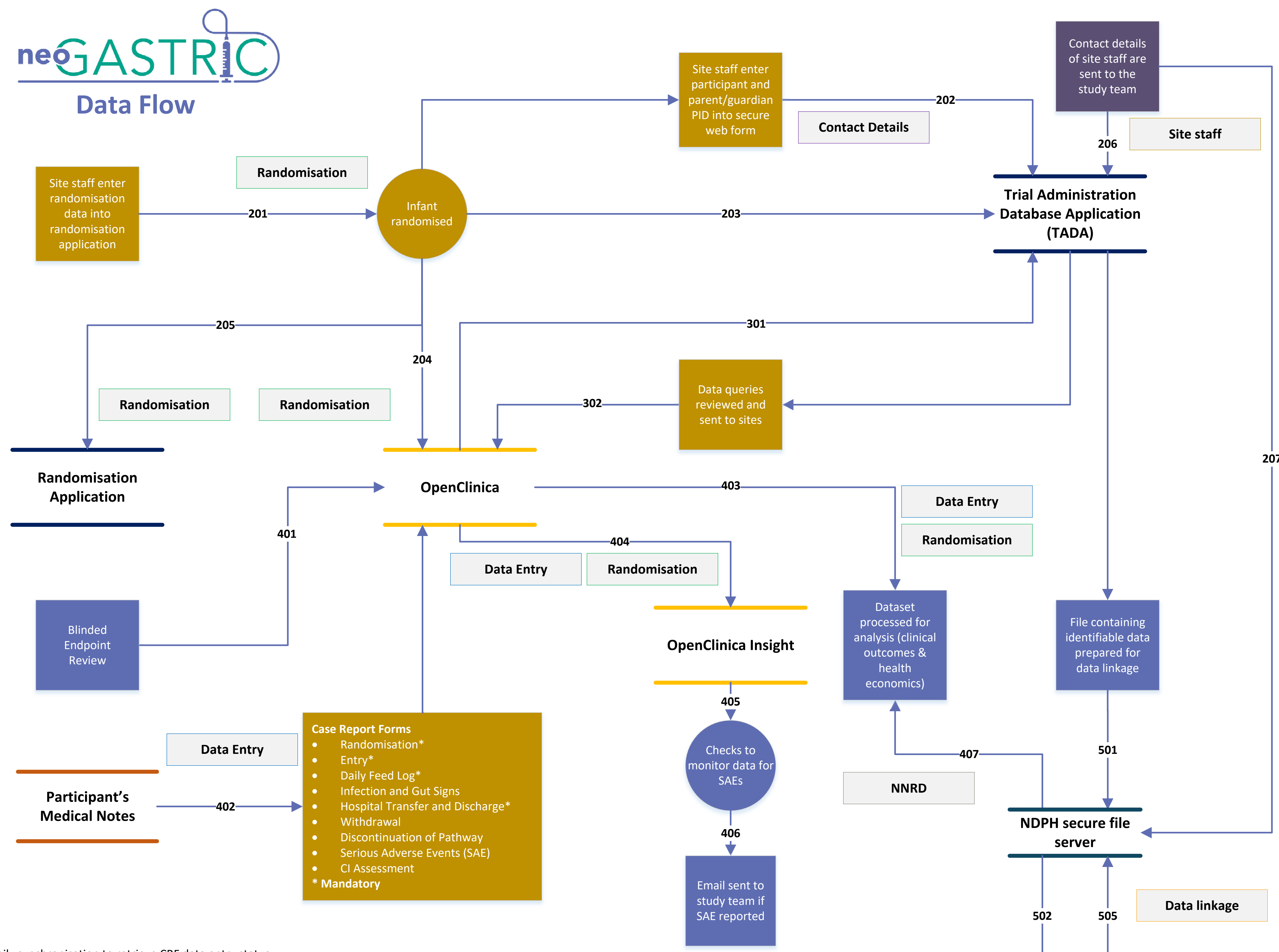

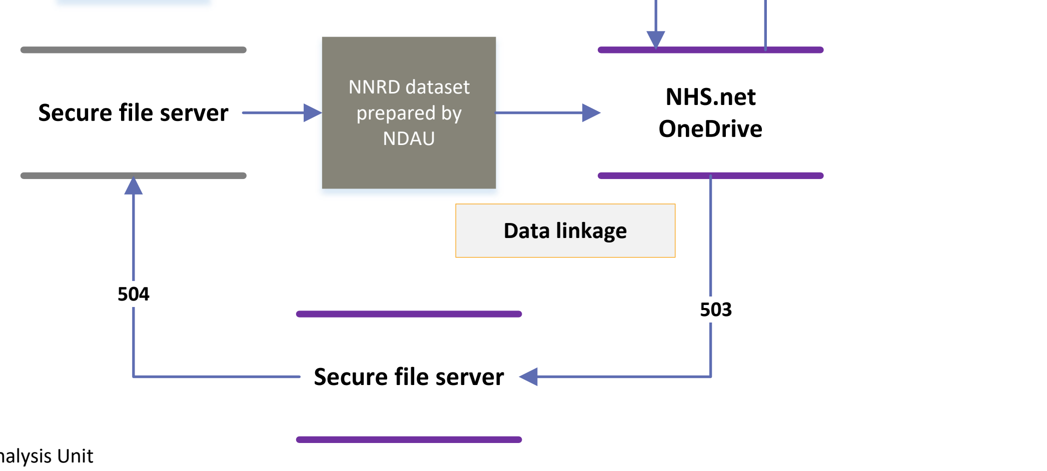

Personal Identifiers (PID)

NNRD

Participant
Ethnicity
Sex
DOB
Postcode (LSOA)

Process Evaluation

Parent
Name
Postal address
Email address
Phone number

Data Entry

Participant
Ethnicity
DOB

Randomisation

Participant
DOB

Contact Details

Participant
Name
Postcode
NHS number (Eng & Wales only)
Badger ID (UK only)
DOB

Data linkage

Participant
NHS Number
Badger/Epic ID

Site staff

Name
Phone number (work)
Email address (work)

Location of data

- University of Oxford (UK) (Controller)
- NHS Sites (UK) (Processor) Medicare Sites (Aus) (Processor)
- University of Liverpool (UK) (Processor)
- Amazon Web Services (UK) (Processor)
- Imperial College (UK) (Controller)
- Chelsea and Westminster NHS Trust (UK) (Processor)

Responsibility Site team | Study team | NDAU

Process Evaluation
101 Paper questionnaire completed by site staff and sent back to UoL
102 Paper questionnaire completed by parent and posted back to UoL
103 Interview with parent completed by UoL

Randomisation
201 Site staff enrol participant using study randomisation application
202 PID transferred using HTTPS protocol and saved into TADA
203 Details of new randomisation POST to TADA
204 Record created in OpenClinica. Randomisation data imported into Randomisation and Entry CRFs
205 Data saved in randomisation MySQL database
206 Details of site staff delegated study duties are entered into TADA
207 Site delegation logs and training records stored sent to NPEU and saved on NDPH file server

TADA
301 Daily synchronisation to retrieve CRF data entry status
302 If required clinical data updated and query closed

OpenClinica
401 Review of NEC and infection case. Result of review entered into OpenClinica
402 Clinical data entered by sites staff into OpenClinica CRFs
403 Dataset exported from OpenClinica and stored in NDPH file server
404 Clinical data and OpenClinica metadata copied into Insight
405 Data monitored for new adverse events
406 Notification email sent to trial team and SAE mailing list
407 NNRD processed for analysis. Datasets stored on NDPH file servers

NNRD
501 Data required for data linkage, saved to restricted area of NDPH file servers; includes PID and unique study number
502 PID dataset uploaded to Imperial College secure data transfer service
503 Data copied from NHS.net OneDrive to ChelWest server and linkage performed
504 Data copied from ChelWest to Imperial College
505 Linked NNRD dataset downloaded from NHS.net OneDrive and saved to secure area on NDPH file server

Abbreviations

NDAU – Neonatal Data Analysis Unit
NNRD – National Neonatal Research Database
NDPH – Nuffield Department of Population Health
