# Supplementary material for: Avoiding routine gastric residual volume measurement in neonatal critical care (the neoGASTRIC trial): study protocol for a multi-centre, unblinded, randomised, controlled trial
Source: Trials. 2026 Jan 8;27:106. doi: 10.1186/s13063-025-09403-7 (PMC12874682; doi:10.1186/s13063-025-09403-7)
Supplement: Supplementary file 4 — Additional file 4. BERC charter. [file 13063_2025_9403_MOESM4_ESM.pdf]

The neoGASTRIC trial: Avoiding routine gastric residual volume measurement in neonatal critical care: a multi-centre randomised controlled trial

ISRCTN registration number: 16710849

REC reference: 23/LO/0060

Chief Investigator: Professor Chris Gale

Australian Chief Investigator: Dr Calum Roberts

Blinded Endpoint Review Committee (BERC) Charter

V2.0 29/01/2025

1. Name and ID of trial

Full title: The neoGASTRIC trial: Avoiding routine gastric residual volume measurement in neonatal critical care: a multi-centre randomised controlled trial

ISRCTN: 16710849

REC reference: 23/LO/0060

Sponsor: Imperial College London

Funder: NIHR Health Technology Assessment programme (HTA)

2. Purpose of Blinded Endpoint Review

A Blinded Endpoint Review Committee (BERC) is a panel of experts with the role of centrally reviewing and classifying endpoints in a blinded and objective fashion, determining whether they fulfil the trial protocol definition in order to harmonise and standardise endpoint assessments. The endpoint review process is intended to enhance the consistency, validity and integrity of the trial's endpoints and/or outcome measures.

3. Scope of charter

This document describes the membership, terms of reference, roles, responsibilities, authority, decision-making and relationships of the Blinded Endpoint Review Committee for data relating to Late Onset Infection and Gut Signs reported on the neoGASTRIC trial. It documents the review process, outcome definitions and review conventions to be used during endpoint review.

4. Qualifications and remit of Blinded Endpoint Review Committee members

The BERC will be chaired by Professor Chris Gale who will take overall responsibility for the endpoint review process. A schedule of agreed BERC members will be maintained (Table 1), and where additional BERC reviewers are added, a new version of the BERC Charter will be produced that names a BERC reviewer in the schedule of Committee members and is authorised by the Chair prior to the additional reviewers undertaking BERC duties.

BERC reviewers will be medical professionals who are expert in the conditions for which endpoint data is being collected for analysis (necrotising enterocolitis and neonatal infection). Reviewers are not required to be independent of the trial as reviews will be carried out blinded to allocation; the potential for bias in the absence of knowledge of the allocation is considered minimal. Participation in the trial as an investigator at a participating site will not preclude membership of the BERC as a reviewer. Reviewers will not review babies who received care at their hospital. The reviewers will be deemed to have no financial conflicts of interest other than being a trial co-investigator. The reviews performed will be compliant with the Data Protection Act.

Table 1: BERC committee members

**Name**	**Role**
Chris Gale	BERC reviewer/Chair
Calum Roberts	BERC reviewer
Nigel Hall	BERC reviewer
Shalini Ojha	BERC reviewer
Jon Dorling	BERC reviewer
Charles Roehr	BERC reviewer
Brett Manley	BERC reviewer
Rod Hunt	BERC reviewer
Cheryl Battersby	BERC reviewer
Peter Davis	BERC reviewer
Elizabeth Nuthall	BERC facilitator
Amy Hutchison	BERC facilitator

5. BERC scope and workflow

The endpoints to be reviewed by Blinded Endpoint Review Committee are:

- Incidence of microbiologically confirmed or clinically suspected late-onset infection (LOI)
- Incidence of necrotising enterocolitis (NEC)
- Focal intestinal perforation (if not NEC)

These reviews will be carried out in batches until all possible cases of LOI and NEC have been reviewed.

Reviews will be conducted as follows:

1. BERC reviewers will review data for babies where at least one *Late-Onset Infection and Gut Signs* form has been received
2. Forms will not be reviewed until the baby has completed the trial and queries on data required for the review are closed
3. Babies may have more than one *Late-Onset Infection and Gut Signs* form completed. Each of these forms will produce one episode to review
4. All episodes for each baby will be reviewed independently by two different BERC reviewers in the first instance
    - If an exact agreement between both BERC reviewers is reached for all episodes, the review is complete
    - If differences occur, it will be referred to a third BERC reviewer who will make the final decision
5. Reviewers will not review babies recruited at or transferred to their hospital
6. If further information is required to classify an episode this will be requested from the recruiting site and the review process for this participant will be put on hold until data is acquired
7. Any forms received from babies who have subsequently withdrawn from further clinical data collection will be eligible for BERC review, but no further information about these participants will be sourced

5.1 BERC data review

To aid accurate endpoint review, the following data will be reviewed alongside each review:

- Gestational age, birth weight and age in days at start of episode
- The majority of the data on the Late-Onset Infection and Gut Signs form, including age of the baby at the start and end dates of the episode
- Whether the baby has died
- Whether the baby has had any SAEs

The arm of the allocated trial intervention will be blinded to reviewers and any information relating to this will be redacted prior to review.

BERC reviewers will be asked to ascertain the following (or note that there is not sufficient data to ascertain):

For each episode, whether the baby had:

- NEC (and if so what stage)
- Focal intestinal perforation (if not NEC)
- Confirmed sepsis (and organism(s) and site(s) of late-onset infection if so)
- Suspected sepsis (if not confirmed sepsis or NEC)

For each baby, the number of distinct **cases** of each of the above will be determined. (The number of cases may be different from the number of episodes.)

Where there is more than one episode for the baby, the reviewer will also be asked to ascertain key data for the case, such as baby's age at start of case (see Appendix 2 for full list of data items).

The decisions will be documented on one OpenClinica form per baby.

Where a baby only has one episode for review, in most cases this will be carried out remotely. More complex scenarios, such as multiple episodes, will mostly be reviewed at face-to-face meetings.

5.2 Further guidance for BERC reviewers

Further guidance: Late-onset infection (LOI) Definitions:

Microbiologically-confirmed Late-onset Infection
Microbiological culture from blood or CSF sampled aseptically more than 72 hours after birth of any of the following
- potentially pathogenic bacteria (including coagulase-negative Staphylococci species but excluding probable skin contaminants such as diphtheroids, micrococci, propionibacteria or a mixed flora)
- fungi
AND
Treatment for 5 or more days with intravenous antibiotics or antifungals after the above investigation was undertaken. If the infant died, was discharged home, or was transferred to another unit prior to the completion of 5 days of intravenous antibiotics, this condition would still be met if the intention was to treat for 5 or more days.
Do not report urinary tract infection unless there is also a positive blood culture.

Clinically-suspected Late-onset infection
Either - Absence of positive microbiological culture, OR - culture of a mixed microbial flora or of likely skin contaminants (diphtheroids, micrococci, propionibacteria) only
AND
Clinician intent to administer antibiotic treatment or intravenous antifungals for 5 or more days (excluding antimicrobial prophylaxis) for an infant who demonstrates 3 or more of the following clinical or laboratory features of invasive infection which commenced more than 72 hours after birth:

- Increase in oxygen requirement or ventilatory support
- Increase in frequency of episodes of bradycardia, desaturations, or apnoea
- Temperature instability
- Ileus or enteral feeds intolerance and/or abdominal distension
- Reduced urine output to <1 ml/kg/hour
- Impaired peripheral perfusion (e.g. capillary refill time >3 seconds, skin mottling or core-peripheral temperature gap >2 degrees centigrade
- Hypotension (clinician defined as needing volume or inotrope support)
- Irritability, lethargy or hypotonia (clinician-defined)
- Increase in serum C-reactive protein levels to >15mg/l or procalcitonin
- White blood cells count <4 or >20 × 10^9 cells/l
- Platelet count < 100 × 10^9 /l
- Glucose intolerance: blood glucose <40 mg/dl [2.2 mmol/l] or >180 mg/dl [10 mmol/l]
- Metabolic acidosis: base excess (BE) <-10 mmol/l or lactate >2 mmol/l

If the infant died, was discharged home, or was transferred to another unit prior to the completion of 5 days of intravenous antibiotics, this condition would still be met if the intention was to treat for 5 or more days.

Further information for BERC reviewers:

- If fewer than three clinical features are reported, this will have been queried with reporting sites as required.
- Where fewer than 3 clinical features are confirmed, and in the absence of microbiological confirmation, the episode will be defined as <u>not LOI.</u>
- In the absence of microbiological confirmation, episodes should be classed as not LOI where less than 5 days antibiotic treatment was given or intended unless the baby died during antibiotic treatment course.
- Regarding “mixed flora”:

- If the mixed flora includes what is considered a definite pathogen (e.g. Gram negative bacillus, enterobacter, S.aureus, GBS, Candida spp., etc.), class as a potentially pathogenic bacteria even if (i) another pathogen is also grown, (ii) a CoNS/Staph epi or similar also grown, or (iii) one of the micro-organisms we define as non-pathogens (diphtheroids, micrococci, propionibacteria) is grown
- Potentially pathogenic organisms (e.g. E.Coli, S.Aureus) are counted as potentially pathogenic bacteria even if part of a mixed growth
- If the mixed flora does NOT include a potentially pathogenic bacteria, but did have a CoNS plus a non-pathogen, or have >1 species of CoNS, then this case should be classed as not LOI
- Please bear in mind that clinicians *and* laboratories may report this differently and may report any pathogenic bactereamia regardless of associated CoNS or non-pathogens

Further guidance: Necrotising Enterocolitis (NEC)

Necrotising enterocolitis:
NEC may be diagnosed at surgery, at post-mortem examination or clinically and radiologically.
Infants who satisfy the definition of NEC below but are found at surgery or post-mortem examination for that episode to have a "Focal Intestinal Perforation" should be coded as having "Focal Intestinal Perforation", not as having NEC.

Bell stage Signs	Systemic	Gastro-intestinal	Radiographic
**Stage IIA**<br>(*Definite NEC: mildly ill)*	Increased desaturations and/or bradycardia<br>Temperature instability<br>Lethargy	Definite abdominal distension<br>Possible abdominal tenderness<br>Possibly bloody stools	Definite abdominal dilatation<br>Pneumotosis intestinalis
**Stage IIB**<br>*(Definite NEC: moderately ill)*	As **IIA** with platelets <100 x 10^{12} and/or metabolic acidosis: base excess <-8 meq/l	Abdominal distension with definite tenderness<br>Possible abdominal wall oedema and/or erythema	As **IIA** with portal vein gas<br>Possible ascites
**Stage IIIA**<br>*(Advanced NEC: bowel intact)*	As **IIB** plus mixed acidosis: pH <7.2<br>DIC neutropaenia <1x10^9/l<br>Severe apnoea<br>Hypotension requiring inotropes	Generalised peritonitis with severe tenderness with abdominal wall induration	As **IIA** with definite ascites
**Stage IIIB**<br>*(Advanced NEC: bowel perforated)*	As **IIIA**	As **IIIA**	As **IIIA** with pneumoperitoneum

Further guidance: Hierarchy and overlapping episodes

- If baby has reported both suspected and confirmed episodes of LOI for the same time intervals, please report confirmed LOI only overall.
- If baby has reported NEC and suspected LOI and you feel the suspected LOI was actually NEC, please report NEC only overall.
- Babies can have both confirmed LOI and NEC/FIP at the same time if this is clearly shown from the data provided.

6. Other possible reviews to be completed by the BERC committee

Primary endpoint: time to full feeds

In some babies, it may not be clear (from the Feed Log data) when full feeds was reached and further data relating to this cannot be obtained from the site or the NNRD. If this occurs, the BERC reviewers may be asked to review these cases. The process will be added to this charter if required.

7. Appendices

Appendix 1: Relevant and blank CRFs appended for reference purposes

Appendix 2: Assessment Form completed by the BERC
